# Occupational stress and work engagement among primary healthcare physicians: a cross-sectional study

**DOI:** 10.1590/1516-3180.2021.0644.R1.10012022

**Published:** 2022-09-12

**Authors:** Luciano Garcia Lourenção, Paula Canova Sodré, Cláudia Eli Gazetta, Albertina Gomes da Silva, Jussara Rossi Castro, José Victor Maniglia

**Affiliations:** I PhD. Nurse and Titular Professor, Postgraduate Nursing Program, Universidade Federal do Rio Grande (FURG), Rio Grande (RS), Brazil.; II PhD. Nurse and Municipal Coordinator of Primary Care Services, Faculdade de Medicina de São José do Rio Preto (FAMERP), São José do Rio Preto (SP), Brazil.; III PhD. Nurse and Adjunct Professor, Faculdade de Medicina de São José do Rio Preto (FAMERP), São José do Rio Preto (SP), Brazil.; IV MSc. Nurse, Faculdade de Medicina de São José do Rio Preto (FAMERP), São José do Rio Preto (SP), Brazil.; V MSc. Dentist, Faculdade de Medicina de São José do Rio Preto (FAMERP), São José do Rio Preto (SP), Brazil.; VI PhD. Physician and Adjunct Professor, Faculdade de Medicina de São José do Rio Preto (FAMERP), São José do Rio Preto (SP), Brazil.

**Keywords:** Primary health care, Physicians, Occupational stress, Work engagement, Occupational health, Family health strategy, Work environment, Unified health system

## Abstract

**BACKGROUND:**

Brazil’s Family Health Strategy is based on a primary healthcare model, which is considered to have case resolution capacity, with physicians at its center.

**OBJECTIVES:**

To evaluate the levels of occupational stress and work engagement among primary healthcare physicians.

**DESIGN AND SETTING:**

Cross-sectional study conducted in 2017, in São José do Rio Preto, São Paulo, Brazil.

**METHODS:**

A non-probability sample including 32 physicians from family health teams was used. Three self-applied instruments were used: a scale developed by the researchers seeking sociodemographic and professional variables, the Work Stress Scale and the Utrecht Work Engagement Scale.

**RESULTS:**

Female professionals (59.4%), permanent employees (56.3%), workload of 40 hours per week (59.4%) and 3-10 years of acting in primary care (68.8%) were more prevalent. Six professionals (19.4%) exhibited significant stress (score ≥ 2.5). The main stressors were lack of prospects for career growth (2.9 ± 1.3), form of task distribution (2.7 ± 1.0), poor training (2.7 ± 1.2) and insufficient time to perform the job (2.6 ± 1.2). Levels of work engagement ranged from 4.3 to 4.6 and were rated as high in all dimensions. Physicians with occupational stress had average levels of work engagement, whereas those without occupational stress had high levels of work commitment.

**CONCLUSIONS:**

A notable percentage of the physicians were experiencing occupational stress. The physicians had high levels of work engagement. Occupational stress was negatively correlated with work engagement, and it significantly compromised physicians’ levels of work engagement and interfered with their positive relationship with the work environment.

## INTRODUCTION

Despite recent advances in primary healthcare in Brazil, numerous difficulties arising from the new National Primary Healthcare Policy continue to permeate the system, thus especially weakening management of workers. The process of recomposition of teams and reorganization of the work process has made it even more difficult to acquire aptly qualified professionals for family healthcare teams, implement more democratic and participatory work processes and regularize contractual bonds. These roadblocks impact employee satisfaction with the work environment and process, and often lead to psychological distress, which then leads to turnover of medical professionals especially.^1–4^

Therefore, it is important to know the level of work engagement among primary healthcare professionals, especially physicians, whose high turnover compromises consolidation of teams and the case resolution capacity of primary healthcare services.^1–4^ Work engagement is considered essential for a good relationship between workers and their company. Engagement is conceptualized as a positive mental state that allows workers to connect deeply with the work activity, and it acts as an indicator of worker health, defined in terms of motivation and professional commitment.^2,5,6^

Engagement involves commitment to the activity and to the work environment and is characterized by three attributes: dedication, absorption and vigor. Dedication comprises the worker’s level of involvement and enthusiasm, manifested as feeling proud and inspired to perform the work. Absorption relates to focus and concentration on the work, which is seen as highly pleasurable. Vigor refers to the level of energy and resilience in the face of adversity.^7,8,9^

Studies conducted in Brazil and elsewhere have indicated that primary healthcare professionals, especially physicians and nurses,^7,10–12^ generally have good levels of engagement at work. A study carried out in two Brazilian cities showed that professionals working in cities with 100% coverage by the Family Health Strategy presented significantly higher levels of engagement than those working in cities with only partial coverage.^10^ These results emphasize the importance of assessing worker engagement as a workforce indicator within primary healthcare.

Likewise, it is important to identify the levels of occupational stress and the stressors related to the working process, in order to obtain the requisite information for reorganizing services and improving working conditions. Such endeavors can contribute towards the productivity and case resolution capacity of workers in the primary healthcare sector and can help retain doctors as well.^1,3,4^

Occupational stress results from conflict between psychological needs and levels of control over the work process. It may arise when the worker, due to lack of training, excessive demand, work overload or precarious safety conditions at work, faces difficulty in coping with challenging situations.^13^

Though at varying degrees, psychological distress is present across all categories of primary healthcare professions. However, there is evidence that physicians are more susceptible to stress due to the high physical and emotional demands that their practice imposes, especially in some specialties such as family and community medicine. Among the factors that cause mental illness among these workers, the most important are those associated with work, such as overload, precarious work conditions, lack of autonomy and pressure to meet targets.^14,15^

Some studies on occupational stress among primary healthcare professionals have highlighted the following as the main stressors: lack of training, type of control in the work environment, lack of prospects for professional growth and lack of autonomy, appreciation and time to perform the work.^7,16,17^ However, there is a lack of information about occupational stress among primary healthcare physicians and the stressors relating to the work processes of these professionals.

Therefore, knowing the levels of work engagement and occupational stress among physicians can generate support for reorganizing the work process and reducing weaknesses that can cause emotional distress. In this manner, positive relationships between workers and their work activities can be strengthened and the productivity and case resolution capacity of primary healthcare services can be improved.

## OBJECTIVE

To evaluate the levels of occupational stress and work engagement among primary healthcare physicians.

## METHODS

### Ethical considerations

Ethical approval regarding this study was obtained from our institutional ethics committee (decision: 1,776,737; date: October 17, 2016). All the participants in this study were only included after written informed consent had been obtained from them. All procedures performed in this study were compatible with the ethical standards of the institutional research committee and with those of the Declaration of Helsinki and its comparable ethical standards.

### Type of study

A quantitative and observational cross-sectional study was conducted in 2017, using a non-probability convenience sample that included 32 physicians from family health teams in São José do Rio Preto, São Paulo, Brazil.

### Sample participants

São José do Rio Preto is a large municipality located in the northwest of the state of São Paulo, 452 km from the state capital. It is the headquarters of the 15^th^ Regional Health Department, the largest in the state, and forms a reference point for healthcare. At the time of this study, the estimated population of this municipality was 446,649 inhabitants and, organizationally, it was divided into five healthcare districts. It had 27 primary care units, consisting of 10 basic health units and 17 basic family health units, with 40 family health teams and 30.9% coverage of the population.

### Setting

The study population comprised all physicians in the family health teams, totaling an estimated 40 professionals. Professionals who were on vacation and/or away from their professional activities during the data collection period were excluded. The sample was defined according to convenience and was composed of 32 physicians (80.0%) who provided responses in the instruments.

### Procedures, measurements, variables and outcome

For data collection, the researchers used a self-administered instrument that investigated sociodemographic and professional variables, and two scales: the Work Stress Scale, validated for use in Brazil by Tamayo and Paschoal;^18^ and the Utrecht Work Engagement Scale, validated for use in Brazil by Vazques et al.^19^

The Work Stress Scale is composed of 23 negative statements to which responses are given using a five-point Likert scale format, ranging from “strongly disagree” to “strongly agree;” the higher the score is, the higher the stress also is. Its indicators were developed from an analysis of the literature on organizational stressors of psychosocial nature and on psychological reactions to occupational stress. The scale has satisfactory psychometric characteristics and can contribute to investigating and diagnosing the organizational environment. It is a tool for organizational diagnosis that has undergone psychometric testing and requirements.^18^

The Utrecht Work Engagement Scale contains 17 self-assessment items grouped into three categories (dedication, absorption and vigor) and an overall score.^19^ These categories are measured as follows:

- Dedication is measured through the average of five items that refer to a sense of meaning, enthusiasm, pride and inspiration for one’s work: 1. I find the work that I do to be full of meaning and purpose; 2. I am enthusiastic about my work; 3. My work inspires me; 4. I am proud of the work I do; and 5. I find my work challenging.^19^
- Absorption is measured through six items that relate to immersion and attachment to work: 1. “Time flies” when I am working; 2. When I am working, I forget everything around me; 3. I feel happy when I work intensely; 4. I feel involved in the work I do; 5. I “get carried away” with my work; and 6. It is difficult to disconnect from work.^19^
- Vigor is measured through six items that relate to energy, resilience, effort and persistence with work: 1. At my work, I feel full of energy; 2. At work, I feel strong and vigorous (vitality); 3. When I get up in the morning, I feel like going to work; 4. I can continue working for long periods of time; 5. I am a mentally resilient person; and 6. At work, I am persistent, even when things are not going well.^19^

Data collection was scheduled by unit managers and was carried out during team meetings. The researchers presented the study objectives, collected the signatures on the informed consent form and handed out the questionnaires. After completion, the questionnaires were deposited in a brown envelope without identification, in order to preserve the participants’ anonymity.

The data were analyzed using the SPSS software, version 23.0, developed by the International Business Machines Corporation (IBM) (New York, United States). Sociodemographic and professional variables were used to describe the physicians’ profiles.

### Sample size and statistical analysis

To analyze occupational stress, a general average score and an average score for each item of the scale were calculated in order to identify the most recent stressors, according to the physicians. The scores could range from one to five, and the higher the average value was, the higher the level of stress was. Mean values greater than or equal to 2.5 indicated higher levels of stress.^18^

The statistical model proposed in the preliminary manual of the Utrecht Work Engagement Scale was used to analyze the levels of engagement at work. The means and standard deviations of the dimensions of the Utrecht Work Engagement Scale were presented.^20^ These dimensions were obtained as follows: Dedication - arithmetic mean of the responses to questions 2, 5, 7, 10 and 13; Absorption - arithmetic mean of the responses to questions 3, 6, 9, 11, 14 and 16; and Vigor - arithmetic mean of the responses to questions 1, 4, 8, 12, 15 and 17. The overall score was obtained through the arithmetic mean of the answers to all questions in the scale.

After the scores had been calculated, the values obtained were interpreted as prescribed in the preliminary manual of the Utrecht Work Engagement Scale: 0 to 0.99 = very low; 1 to 1.99 = low; 2 to 3.99 = medium; 4 to 4.99 = high; and 5 to 6 = very high.^20^

The internal consistency indicator Cronbach’s alpha was used to ascertain the reliability of the measurements of the constructs of the Utrecht Work Engagement Scale. Comparison of the mean scores of the scales was performed using the t test for two means or using analysis of variance, with a significance level of 95% (P ≤ 0.05).

The correlation between occupational stress and work engagement was assessed using Pearson’s correlation test. In this, r up to 0.30 was taken to represent a weak correlation; r between 0.40 and 0.60, moderate correlation; and r greater than 0.70, strong correlation.

## RESULTS

Thirty-two physicians working at primary healthcare units participated in this study; 19 (59.4%) were female and 20 (62.5%) were married. Their ages ranged from 27 to 75 years, with a mean age of 45.2 years and standard deviation (SD) of 11.7 years.

As shown in Table 1, 15 (46.9%) of these professionals had undergone specialized training, 17 (53.2%) were overweight or obese, 18 (56.3%) were permanent employees, 19 (59.4%) worked at the primary care unit for 40 hours a week, 16 (50.0%) were involved in another paid activity, 16 (50.0%) practiced physical activity, 25 (78.1%) reported engaging in leisure activities, 17 (53.1%) practiced religious observance and 23 (71.9%) had six to eight hours of sleep per night. The monthly family income reported by 22 physicians (68.8%) was higher than 10 minimum monthly wages (the current minimum monthly wage value is R$ 937.00, i.e. approximately US$ 284.00). The length of employment of these professionals working in primary healthcare ranged from six months to 30 years, with a median of seven years.

**Table 1.** Sociodemographic characteristics of primary healthcare physicians

Variables	n	%
**Gender**		
Male	13	40.6
Female	19	59.4
**Age group**		
Up to 30 years old	3	12.5
From 31 to 49 years old	16	50.0
50 years old or over	12	37.5
**Marital status**		
Married	20	62.5
Single	9	28.1
Divorced	2	6.3
Widowed	1	3.1
**Education level**		
Bachelor’s degree	11	34.4
Specialist degree	15	46.9
Master’s degree	5	15.6
Doctoral degree	1	3.1
**Body mass index**		
Normal	11	34.4
Overweight	14	43.8
Obesity grade I	2	6.3
Obesity grade III	1	3.1
No information	4	12.5
**Type of contract**		
Permanent (statutory regime)	18	56.3
Contracted (consolidation of Brazilian labor laws)	14	43.8
**Weekly workload**		
20 hours	9	28.1
30 hours	4	12.5
40 hours	19	59.4
**Family income** (minimum wages)^*^		
From 6 to 10 minimum wages	9	28.1
More than 10 minimum wages	22	68.8
No information	1	3.1
**Other remunerated activity**		
Yes	16	50.0
No	16	50.0
**Practice of physical activity**		
Yes	16	50.0
No	16	50.0
**Recreational activity**		
Yes	25	78.1
No	7	21.9
**Frequent religious observance**		
Yes	17	53.1
No	15	46.9
**Daily hours of sleep**		
Less than 6 hours	9	28.1
From 6 to 8 hours	23	71.9

* Minimum monthly wage value: R$ 937.00 (US$ 284.00).

In the occupational stress analysis, one professional was excluded because of not having answered the questions of this instrument. The general average obtained among the 31 physicians evaluated was 2.1, with a SD of 1.1. It was observed that eight (25.0%) of the professionals presented scores that corresponded to major stress (≥ 2.5).

As can be seen in Table 2, the major stressors were lack of prospects for career growth (2.9; SD = 1.3), the way in which tasks were distributed (2.7; SD = 1.0), deficiencies in professional training (2.7; SD = 1.2), insufficient time to perform the job (2.6; SD = 1.2), the type of control imposed (2.5; SD = 1.0) and lack of autonomy in executing the job (2.5; SD = 1.2).

**Table 2.** Rating of the items of the Work Stress Scale, according to the perceptions of the primary healthcare physicians

Items	Mean (± standard deviation)
**Q1** - The way tasks are distributed in my area makes me irritated	**2.7 (1.0)**
**Q2** - The kind of control that exists in my work annoys me	**2.5 (1.0)**
**Q3** - The lack of autonomy in implementing my work is exhausting	**2.5 (1.2)**
**Q4** - I am uncomfortable with my superior’s lack of confidence in my work	1.8 (1.0)
**Q5** - I am irritated by the lack of disclosure of information about organizational decisions	2.4 (1.2)
**Q6** - I feel uncomfortable with the lack of information about my tasks at work	2.1 (1.0)
**Q7** - Lack of communication between my coworkers and me makes me angry	2.1 (0.9)
**Q8** - I feel annoyed that my superior mistreats me in front of coworkers	1.5 (0.7)
**Q9** - I feel uncomfortable having to perform tasks that exceed my capacity	2.0 (1.1)
**Q10** - I get in a bad mood through having to work for many hours at a time	2.4 (1.2)
**Q11** - I feel uncomfortable with the communication between my superior and me	1.8 (1.1)
**Q12** - I get irritated with discrimination/favoritism in my work environment	1.9 (1.0)
**Q13** - I am uncomfortable with the deficient professional training	**2.7 (1.2)**
**Q14** - I get in a bad mood because I feel isolated in the organization	2.1 (1.0)
**Q15** - I get annoyed at being undervalued by my superiors	2.1 (1.3)
**Q16** - The few prospects for career growth make me distressed	**2.9 (1.3)**
**Q17** - I am uncomfortable about working on tasks below my skill level	2.4 (1.3)
**Q18** - The competition in my work environment puts me in a bad mood	1.5 (0.6)
**Q19** - Lack of understanding of what my responsibilities are in this work annoys me	2.0 (1.0)
**Q20** - I get irritated about my superior giving me contradictory orders	1.6 (0.8)
**Q21** - I feel annoyed that my superior is covering up my well-done job in front of other people	1.6 (0.8)
**Q22** - Insufficient time to carry out my workload makes me irritated	**2.6 (1.2)**
**Q23** - I am annoyed that my superior prevents me from taking on significant responsibilities	1.7 (0.8)

Values in bold indicate items of the Work Stress Scale with scores compatible with a significant level of occupational stress.

The levels of physicians’ work engagement are presented in Table 3. In the reliability analysis, Cronbach’s alpha coefficient values ranged from 0.833 to 0.950, thus indicating that the results showed good reliability. The means of the dimensions ranged from 4.3 (SD = 1.2) to 4.6 (SD = 1.3). Both dimensions presented engagement levels classified as high (Table 3).

**Table 3.** Levels of engagement shown in the Utrecht Work Engagement Scale among the primary healthcare physicians

Dimensions	Cronbach’s alpha	Minimum	Maximum	Median	Mean (± SD)	95% CI	Interpretation of level of engagement
**Dedication**	0.922	0.6	6.0	4.7	4.6 (1.3)	4.1–5.0	High
**Absorption**	0.833	1.5	6.0	4.5	4.3 (1.2)	3.9–4.7	High
**Vigor**	0.884	1.8	6.0	4.9	4.5 (1.1)	4.1–4.9	High
**General score**	0.950	2.0	6.0	4.7	4.5 (1.1)	4.1–4.9	High

SD = standard deviation; CI = confidence interval.

Occupational stress and work engagement correlated negatively (Table 4). We observed that the correlation between occupational stress and the attributes of dedication (r: -0.357; P = 0.049) and absorption (r: -0.369; P = 0.041) was weak, while it was moderate between occupational stress and vigor (r: -0.444; P = 0.012) and overall (r: -0.519; P = 0.003).

**Table 4.** Correlations between the Work Stress Scale and the Utrecht Work Engagement Scale

Work Engagement Scale dimensions	Work Stress Scale	P value*
**Dedication**	-0.357**	**0.049**
**Absorption**	-0.369**	**0.041**
**Vigor**	-0.519***	**0.003**
**General score**	-0.444**	**0.012**

* t test; **P < 0.05; ***P < 0.01.

From analysis on the levels of engagement, according to the presence or absence of occupational stress (Table 5), we observed that physicians demonstrating occupational stress showed average levels of engagement in relation to all the parameters of the Utrecht Work Engagement Scale. On the other hand, physicians who did not demonstrate any occupational stress showed high levels of engagement in relation to all the parameters. This analysis confirmed that occupational stress compromised the positive relationship of these professionals with their work environment.

**Table 5.** Analysis on the levels of work engagement among the primary healthcare physicians, according to the presence or absence of occupational stress

Work engagement	Occupational stress	Mean score	Standard deviation	Interpretation of levels of engagement	P value*
**Dedication**	Yes	3.71	0.94	Medium	**0.010**
	No	4.90	1.30	High	
**Absorption**	Yes	3.46	1.12	Medium	**0.006**
	No	4.66	1.00	High	
**Vigor**	Yes	3.37	1.07	Medium	**< 0.001**
	No	4.97	0.74	High	
**General score**	Yes	3.50	0.99	Medium	**0.001**
	No	4.84	0.94	High	

* t test.

## DISCUSSION

The sociodemographic profile of the physicians in this study corroborates the findings from other studies conducted in Brazil and elsewhere.^7,15,21,22,23,24^ Currently, in the field of medicine, there is a predominance of women working in various positions, including under precarious working conditions, thus leading to high occupational stress.^25^

The percentage of these physicians presenting significant occupational stress corroborated data in the literature on this topic published in Brazil and elsewhere.^7,26,27^ This showed that organizational stressors interfered with practice among the physicians evaluated. These stressors may have arisen through the structure and political-administrative organization of primary healthcare within the municipality. At the time of this study, the Family Health Strategy covered 30.9% of this city’s primary care. Large cities have generally implemented the Family Health Strategy in poorer regions that present greater demands for healthcare and generate greater challenges and workload for the professionals, thereby increasing their risk of developing occupational stress.^10^

As shown in this study, the most stressful factor in the physicians’ perception was lack of career growth prospects. This was a major obstacle in retaining physicians within primary healthcare services, especially in remote areas.^3,4^ Moreover, lack of career advancement opportunities may play an important role in occurrences of exhaustion among primary healthcare providers.^28^ This result highlights the need for creation and implementation of a medical career path within Brazilian primary healthcare services, at both the state and the federal level.

Another stressor was deficiency of professional training. This compromises job satisfaction and has a high correlation with professional turnover within primary healthcare.^3^ A study among primary healthcare teams in a small city in the interior of the state of São Paulo that has 100% coverage by the Family Health Strategy indicated that lack of training was the main cause of occupational stress, thus corroborating the results from the present study.^7^

Moreover, task distribution, insufficient time to perform the work, type of control and lack of autonomy in performing the work were found to be associated with decreased levels of job satisfaction and increased stress. These factors can culminate in development of burnout syndrome among physicians and other professionals within primary healthcare services.^26,29,30^

The presence of occupational stress among physicians who did not practice any religious observance that was found in the present study corroborated the findings of a previous study.^27^ However, it is noteworthy that family physicians have a higher risk of developing stress and emotional distress, regardless of sociodemographic factors, compared with other specialties.^26^ In this context, it is essential that municipal managers are aware of the aspects of the work process within primary healthcare that can cause occupational stress, given that protection and development of family physicians’ health will have a positive impact on the health of all professionals and on the quality of care provided for users.^31^

Furthermore, identification and correction of the causal factors behind occupational stress among physicians can reduce burnout and favor work involvement.^28^ This will promote compliance with the guidelines of the new National Primary Care Policy,^32^ especially with regard to continuity of care, retention of physicians, establishment of bonds and appropriate accountability of professionals and users.^14^ Through this, primary healthcare services can be strengthened.

The primary healthcare physicians in the present study showed higher levels of work engagement than those reported in previous Brazilian studies on healthcare professionals undergoing training,^33^ professionals within multiprofessional residency programs^9^ and military police officers.^34^ However, these results corroborated the findings of Brazilian studies on other primary healthcare professionals^7,11,16^ and those of studies conducted in other countries among dentists,^35^ primary healthcare nurses^36^ and hospital care nurses,^37^ thus indicating that physicians present high levels of work engagement in the primary healthcare sector.

High levels of work engagement among physicians are important for the healthcare system, since these indicate that these professionals can contribute very positively towards meeting the needs of the enrolled population. High levels of energy and connection with their work allow them to cope better with the demands of their practice.^9,38^

However, it has been observed that occupational stress significantly compromises the levels of engagement with work among primary healthcare physicians, which can negatively affect the work performance of these professionals.^7^ Moreover, this stress is detrimental to these professionals’ willingness to continue working in primary healthcare teams.^4^

Since work engagement is related to workers’ involvement with the work activity and professional effectiveness, occupational stress will also compromise the level of physicians’ wellbeing, thus leading to demotivation and dissatisfaction with the work.^39^ Therefore, implementation of positive managerial policies that promote recognition and appreciation can stimulate these professionals’ engagement. Above all, this improves their resilience to work adversities and avoids the negative impact of stressors on their engagement levels.^7,40^ It is noteworthy, however, that the responses to positive or negative stimuli may vary between different regions and healthcare units because engagement is a phenomenon that is associated with the team in which the physician is placed, as shown in a study among Portuguese nurses, whose levels of engagement varied between different regions, hospitals and care units.^41^

It is noteworthy that shared management can favor increased engagement among primary healthcare professionals through enabling participation in decision-making, thereby supporting the work process and strengthening team relationships. Likewise, allowing involvement of all workers can facilitate overcoming individual and collective difficulties, thus reducing occupational stress.^42,43^

The main limitations of this study were its cross-sectional design, which made it impossible to establish cause-and-effect relationships; and its inclusion of professionals from a single municipality, with a limited sample that does not allow generalization of the results. However, this study has provided an important diagnosis for the relationship between the work process and emotional health of physicians who work in primary healthcare services in a large city, thus contributing useful information for improvement of the work process in municipal primary healthcare services.

## CONCLUSION

A notable percentage of the primary healthcare physicians surveyed presented occupational stress. The major stressors, in these professionals’ perception, were lack of prospects for career growth, the way in which tasks were distributed, deficiencies in professional training, insufficient time to perform the work, the type of control imposed and lack of autonomy in performing the work.

The physicians showed high levels of work engagement, which showed that they had energy and willingness to work; they were proud, enthusiastic, focused and persistent in the face of adversity in the work environment. Occupational stress and work engagement were negatively correlated, and occupational stress significantly compromised the physicians’ levels of work engagement.
